# Antinociceptive Effect of Ficus bengalensis Bark Extract in Experimental Models of Pain

**DOI:** 10.7759/cureus.2259

**Published:** 2018-03-02

**Authors:** Kartikeya Rajdev, Seema Jain, Mahendra C.H, Swapan K Bhattacharaya

**Affiliations:** 1 Internal Medicine, Staten Island University Hospital, Northwell Health; 2 Department of Pharmacology, University College of Medical Sciences and Gtb Hospital, New Delhi, India-110095; 3 Professor/department of Pharmacology, Bada Hindu Rao Hospital, New Delhi, India-110007

**Keywords:** animal models, antinociceptive, analgesic, tail flick test, ficus bengalensis, formalin test

## Abstract

The present study was carried out to study the effect of the aqueous extract of the stem bark of Ficus bengalensis (F. bengalensis) Linn on animal models of pain. The study consisted of five groups of Swiss albino mice with eight mice in each group. The first group served as the control group and received only normal saline. The second, third, and fourth groups received the aqueous extract of F. bengalensis per orally (p.o.) in doses of 100, 200, and 400 mg/kg respectively. The last group received morphine intraperitoneally. The antinociceptive effect was studied using the tail-flick and formalin-induced pain tests. In the tail-flick test, a significant increase in the time elapsed till the animal flicked its tail from the source of thermal stimuli was obtained with all three tested doses of F. bengalensis extract as compared to the control group. In the early phase of the formalin test (0-5 min), a significant decrease in the duration of licking response was observed with F. bengalensis (at doses of 200 and 400 mg/kg) and morphine-treated groups as compared to the control group. In the late phase (25-30 min) of the formalin test, the duration was significantly reduced with all three treated doses of F. bengalensis when compared with the control group. A similar result was obtained with the morphine-treated group. The results indicate that F. bengalensis extract possesses significant antinociceptive activity in the mice.

## Introduction

Plants, as illustrated throughout the history of civilization, have served as a major source of medication for the treatment of human ailments. Medicinal plants are being increasingly used in both developing and developed countries not only as herbal medicines but also as dietary supplements. They contain many active constituents that are effective in the treatment of many human diseases. A large number of populations rely on herbal medicines because of their ethnic acceptability and compatibility, fewer side effects, and relatively low cost [1-2]. Although herbal medicines have existed since time immemorial, our knowledge of how plants actually affect human physiology remains largely unsettled. Medicinal plants have provided the basis for the discovery of many bioactive compounds that served and continue to serve as the lead molecule for the development of new drugs. F. bengalensis is one such medicinal plant. Ficus Linn (Family: Moraceae), with about 800 species of free-standing trees, hemiepiphytes, and shrubs, constitutes one of the largest genera of flowering plants primarily occurring in subtropical and tropical regions worldwide.

F. bengalensis Linn, commonly known as the Banyan tree or Vata or Vada, has a prominent place in Hindu religions and Ayurvedic medical literature. It is considered sacred in many places in India. F. bengalensis is reported to have several therapeutic uses in folk medicine [3]. Its bark has been used for the treatment of diarrhea and dysentery, and its latex has been used for the treatment of hemorrhagic diarrhea, dysentery, and inflammation [4-5]. All parts of the plant have been used in both the Unani and Ayurveda systems of medicine as astringent, anodynia, anti-inflammatory, anti-arthritic, diaphoretic, antidiarrhoeal, and antiemetic [6-9]. The plant has also been known to possess antimicrobial and immunomodulatory properties [10-11]. The milky juice obtained from the stem, seed, or fruit of the banyan tree is used for the treatment of rheumatism and other inflammatory diseases in Ayurveda in India [9,12]. In experimental studies, the extract of F. bengalensis is also reported to possess antidiabetic, immunomodulatory, hypolipidemic, antioxidant, and antiasthmatic activity [9,13-15]. Throughout history, several forms of therapies have been used for the relief of pain; among them, the use of medicinally active plants has increased in developed and developing countries to promote healing and alleviate pain. As most of the analgesic drugs being used today exert a wide range of side effects, a study on plant species that are traditionally used as painkillers should still be seen as a logical and fruitful research strategy, in search of analgesic drugs. Although many properties of F. bengalensis have been reported in the past, the literature shows lacunae in the study of its analgesic effects, particularly in the background of nociceptive behavior. Nociception or pain is an unpleasant sensation localized to a part of the body. It is often penetrating in nature, or has tissue destructive processes (stabbing, burning, twisting, and squeezing), and having an emotional reaction. The present study was carried out to evaluate the antinociceptive activity of the aqueous bark extract of F. bengalensis in mice by using two methods, the tail-flick test and the formalin test.

## Materials and methods

This study was conducted in the department of pharmacology at University College of Medical Sciences, New Delhi, India. This is an experimental study done on animal models of pain. Two variables have been measured in the study, tail-flick latency and the formalin test.

Drugs and chemicals used in the study

Normal saline; commercially available bark extract of F. bengalensis; and morphine

Animals

Swiss albino mice of either sex, weighing between 25 and 30 grams were taken for the study. Animals were procured from the Central Animal House, University College of Medical Sciences, Delhi. Animals were housed in groups of eight mice per cage with a natural light/dark cycle and provided with free access to pellet diet and water. The study was approved by the Institutional Animal Ethics Committee (IAEC), University College of Medical Sciences, Delhi. Care of animals was taken as per the guidelines of Committee for the Purpose of Control and Supervision of Experiments on Animals (CPCSEA).

Plant Extract

The standardized aqueous bark extract of F. bengalensis was purchased from Tapovan Ayurved Sadan, Najafgarh, New Delhi, India (Member: Central Institute of Medicinal and Aromatic Plants, Lucknow, India). As per the literature provided by the manufacturer, the extract was found to contain flavonoids, terpenoids, tannins, saponins, sugars, glycosides, and phenolic compounds. For the purpose of this study, the F. bengalensis extract was dissolved in double distilled water to prepare suspensions of the required doses of 100, 200, and 400 mg/kg.

Treatment Schedule

The extract was administered once in doses of 100, 200, and 400 mg/kg per orally (p.o.) one hour prior to the experiment, and morphine was administered in a dose of five mg/kg intraperitoneally, 30 minutes (min) prior to the experiment. Normal saline was administered to the control group. All drugs were administered in a volume of 10 ml/kg/dose. The animals were divided into five groups with eight mice in each group. The first group received only normal saline and served as the control group. The second, third, and fourth groups were administered the aqueous extract of F. bengalensis in doses of 100, 200, and 400 mg /kg p.o., respectively 60 min. prior to the experiment while the fifth group was injected with morphine in the dose of five mg/kg, 30 min prior to the experiment.

Tail-Flick Test

The tail-flick test was used in mice to elicit a spinal tail-flick response to noxious thermal stimuli. This test was used to measure the latency of the response as described by D’amour and Smith with modifications [16]. The test was performed with the tail-flick analgesia meter (Ugo Basile, Italy), consisting of an infrared radiant light source. Each mouse was gently held with one hand and its tail was positioned on the source of radiant heat. When the animal flicked its tail from the point source in response to thermal noxious stimuli, both the heat source and the timer were automatically stopped. The time elapsed (in seconds) till the animal flicked its tail was determined. The test was done at 60 min. after the administration of F. bengalensis extract and 30 min. after morphine administration. A cut-off time of 10 seconds was used to prevent tissue damage.

Formalin Test

The formalin test was done as described by Abbott et al. [17]. Mice were injected with 0.05 ml of one percent formalin into the subplantar surface of the right hind paw. The left hind paw was injected normal saline and was considered the control. Animals were immediately returned individually into the testing chamber. The nociceptive response was quantified as the duration (in seconds) of licks and bites of the injected paw during the early phase (0-5 min) and late phase (25-30 min). Antinociceptive activity of F. bengalensis was assessed using single dose administration. Morphine was given intraperitoneally 30 min. prior and F. bengalensis extract was administered p.o. 60 min. prior to the induction of nociceptive behaviors in the animals.

Statistical Analysis

The results were expressed as mean ± SEM. The data were analyzed statistically using one-way analysis of variance (ANOVA) followed by post hoc Tukey's test. P values less than 0.05 were considered significant. Statistical analysis of data was done using GraphPad Prism (GraphPad Software Inc., California, US) software.

## Results

Effects of F. bengalensis extract on the tail-flick latency test

**Figure 1:**
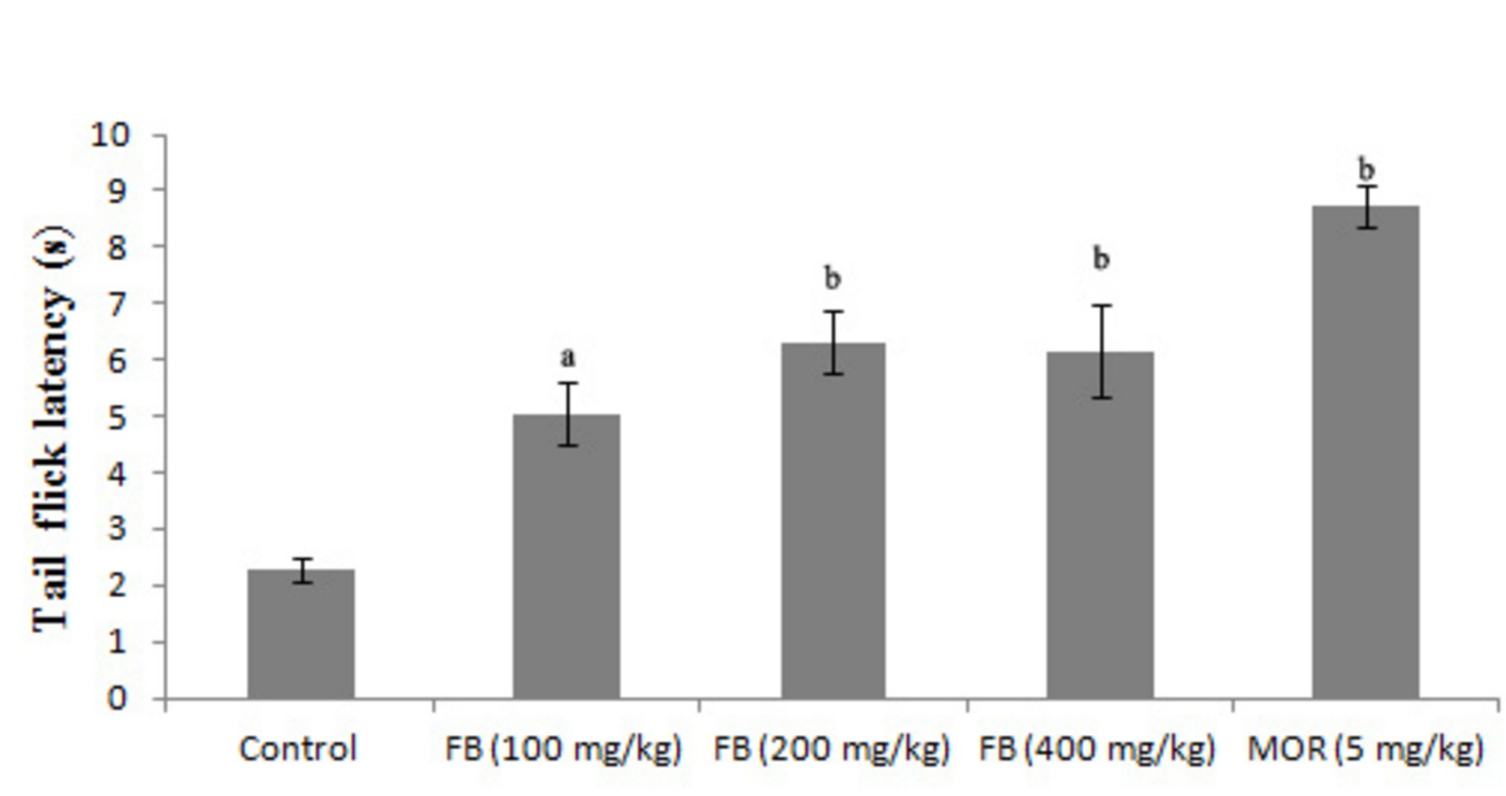
Effects of F. bengalensis bark extract on tail-flick latency in mice (n=eight) F. bengalensis (FB) was administered in doses of 100, 200, and 400 mg/kg p.o. one hour before test. Morphine (MOR) was administered in a dose of five mg/kg, intraperitoneally, 30 min before test. Values are expressed as mean ± SEM (One way ANOVA followed by Tukey’s post hoc test). a denotes p<0.05 while b denotes p<0.001 as compared to the control group.

The effect of the stem bark powder was evaluated in three doses in the tail-flick model of pain in mice. The pretreatment of mice with doses 100 mg/kg (p<0.05), 200 mg/kg (p<0.001), and 400 mg/kg (p<0.001) increased the mean tail-flick latency compared to the control group. Mice treated with morphine (five mg/kg) also showed an increase in tail-flick latency as compared to the control group (p<0.001) (Figure 1).

Effects of F. bengalensis extract on formalin-induced pain response

**Figure 2:**
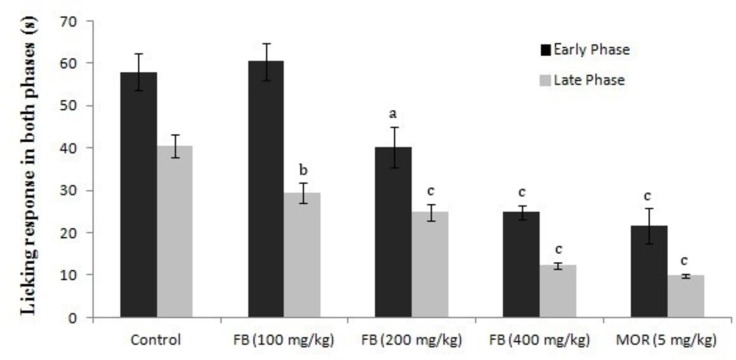
Effects of F. bengalensis bark extract on the early and late phases of formalin-induced pain response in mice (n=eight) F. bengalensis (FB) was administered in doses of 100, 200, and 400 mg/kg p.o. one hour before the test. Morphine (MOR) was administered in a dose of five mg/kg intraperitoneally, 30 min before test. Values are expressed as mean ± SEM (one-way analysis of variance (ANOVA) followed by Tukey’s post hoc test). a denotes p<0.05, b denotes p<0.01, and c denotes p<0.001 as compared to the control group.

Injection of 0.05 ml of one percent formalin into the subplantar surface of the right hind paw induced a pain response that was quantified as the duration of licks and bites. The duration of pain response in the early phase (0-5 min) was significantly reduced in F. bengalensis (200 and 400 mg/kg) and morphine-treated groups as compared to the control group. In the late phase (25-30 min), the duration was significantly reduced with all three treated doses of F. bengalensis when compared with the control group. A similar result was obtained with the morphine-treated group as compared to the control group (Figure 2).

## Discussion

The present study was carried out to investigate the antinociceptive activity of the extract of F. bengalensis using two experimental models of pain, i.e. the tail-flick and formalin-induced pain response tests that employ thermal and chemical-induced nociception stimuli, respectively. These models are commonly used to evaluate central and peripheral analgesic activity of a compound. The tail-flick test, a well-known experimental model of pain, is used to assess the effectiveness of centrally acting analgesics. The analgesic action in tail-flick involves a spinal component and mediates the spinal reflex to painful stimuli [18-19]. In the present study, the oral administration of an aqueous extract of F. bengalensis in doses of 100, 200, and 400 mg/kg significantly prolonged the latency of nociceptive response in the tail-flick test in mice as compared to treatment with normal saline in the control group, suggesting a central analgesic effect of the extract. We can postulate that F. bengalensis exerted its analgesic effect by increasing the animal’s capacity to tolerate pain, which is an unpleasant sensation to part of its body.

The formalin-induced pain response model is used to measure both antinociceptive and anti-inflammatory responses and is more appropriate to measure the response to a long-lasting nociceptive stimulus in contrast to the tail-flick test [20]. The nociceptive behavior in the formalin test is observed in two distinct phases-early and late phases-that indicate different types of pain [21]. The early phase, named noninflammatory pain, corresponds to acute neurogenic pain and occurs as a result of the direct activation of nociceptors, thus reflecting centrally mediated pain. This phase is inhibited by opioid analgesics [22]. The late phase, named inflammatory pain, occurs through the activation of ventral horn neurons at the spinal cord levels. This phase is attributed to the release of various inflammatory and hyperalgesic mediators, such as histamine, serotonin, bradykinins, and prostaglandins. This phase is inhibited by both centrally acting drugs, such as opioids, and peripherally acting drugs, such as cyclooxygenase inhibitors, and has been used to evaluate the effect of nonsteroidal anti-inflammatory drugs (NSAIDs) which primarily inhibit the cyclooxygenase involved in prostaglandin synthesis [23]. In the formalin-induced pain response test, the administration of F. bengalensis extract significantly prolonged the duration of pain response in both the early and late phases of the test. The antinociceptive effect observed in both pain models with F. bengalensis indicates the role of F. bengalensis in peripheral as well as central pain mechanisms. Based on this, it can be inferred that the peripheral analgesic effect observed with the F. bengalensis extract could be due to the inhibition of enzyme COX or any other inflammatory mediators, leading to the inhibition of prostaglandins synthesis.

A few studies on F. bengalensis have demonstrated its role in the hot plate and acetic acid-induced writhing models of pain sensitivity. This study is the first to demonstrate the effect of the aqueous extract of F. bengalensis in the formalin and tail-flick models in mice. Thakre et al. have reported that the administration of a methanol extract of F. bengalensis at doses of 200 mg and 400 mg/kg, p.o. caused a significant reduction in the frequency and time duration of writhing movements in the acetic acid-induced writhing test in mice [9]. Similarly, Patil et al. revealed the analgesic and antipyretic properties of the various extracts from the bark of F. bengalensis using the hot-plate method and the tail-immersion method in rats [24]. The anti-inflammatory effect of F. bengalensis has also been demonstrated in carrageenan-induced paw edema and cotton pellet- induced granuloma models in rats [16].

The extract of F. bengalensis contains various active principles, such as flavonoids, tannins, terpenoids, glycosides, carbohydrates, amino acid, and phenolic compounds. The tannins, terpenoids, and flavonoids isolated from various plant extracts have demonstrated anti-inflammatory and analgesic activity in various studies [25-26]. Different flavonoids molecules have been reported to have an inhibitory effect on various enzymes like phospholipase A2, cyclooxygenase, and lipoxygenase, thus reducing the production of the arachidonic acid, prostaglandins, and leukotrienes that are involved in the late phase of pain perception. They also inhibit other enzymes, such as protein kinase C, protein tyrosine kinases, phosphodiesterases, etc [27-28]. Terpenoids also exert significant analgesic and anti-inflammatory effect by inhibiting phospholipase A2, thereby preventing the synthesis of prostaglandins [29-30]. It is likely that the central and peripheral analgesic effect observed following the administration of F. bengalensis extract may be due to the presence of various phytochemicals, especially flavonoids, tannins, or terpenoids. Our study was aimed at evaluating the analgesic activity of aqueous bark extract of F. bengalensis. However, detailed studies are warranted in this direction to decode the exact nature of the phytochemical compounds responsible for the analgesic effects that could help in developing a drug that is effective and safer than the other analgesics used.

## Conclusions

The findings of our study revealed the antinociceptive effect of F. bengalensis extract in the pain model of the tail-flick and formalin-induced response tests. In conclusion of our findings, we suggest that the aqueous extract of F. bengalensis is a promising analgesic agent and may be useful in the treatment of painful conditions and diseases. However, studies are required on human subjects to prove its clinical efficacy as an analgesic agent.
